# The Severity of Traumatic Stress Associated with COVID-19 Pandemic, Perception of Support, Sense of Security, and Sense of Meaning in Life among Nurses: Research Protocol and Preliminary Results from Poland

**DOI:** 10.3390/ijerph17186491

**Published:** 2020-09-07

**Authors:** Grzegorz Józef Nowicki, Barbara Ślusarska, Kinga Tucholska, Katarzyna Naylor, Agnieszka Chrzan-Rodak, Barbara Niedorys

**Affiliations:** 1 Department of Family Medicine and Community Nursing, Medical University of Lublin, Staszica 6 Str., PL-20-081 Lublin, Poland; basiaslusarska@gmail.com (B.Ś.); agnieszkachrzan607@op.pl (A.C.-R.); baskaniedorys@gmail.com (B.N.); 2 Institute of Applied Psychology, Faculty of Management and Social Communication, Jagiellonian University in Krakow, Lojasiewicza 4 Str., PL-30-348 Krakow, Poland; kinga.tucholska@uj.edu.pl; 3 Department of Didactics and Medical Simulation, Medical University of Lublin, Chodźki 4 Str., PL-20-093 Lublin, Poland; Katarzyna.Naylor@hotmail.com

**Keywords:** COVID-19, nurses, posttraumatic stress disorder, perceived social support, changes in outlook, sense of security, meaning in life

## Abstract

The COVID-19 pandemic can not only affect physical health, but also mental health, resulting in sleep problems, depression, and traumatic stress. Our research investigates the level of posttraumatic stress, perceived social support, opinions on positive and negative consequences of the pandemic, sense of security and sense of meaning among nurses in the face of this new and not fully understood global epidemiological phenomenon. For this purpose, computer-assisted web interviews were conducted between May 1 and May 15, 2020. Participating nurses completed the following research tools: The Impact Event Scale-Revised (IES-R), The Multidimensional Scale of Perceived Social Support (MSPSS), The Changes in Outlook Questionnaire (CIOQ), The Safety Experience Questionnaire (SEQ) and The Meaning in Life Questionnaire (MLQ). Three hundred and twenty-five nurses of an average age of 39.18 ± 11.16 years and working throughout Poland joined the study. The average overall IES-R score in the study group was 1.78 ± 0.65. Among the dimensions of traumatic stress, the highest score was obtained in the “avoidance” dimension was 1.86 ± 0.73. Amongst participating nurses, the highest support rates were provided by significant others (22.58 ± 5.22). Higher average scores were noted among participants in the subscale measuring positive psychological changes (18.56 ± 4.04). The mean MLQ score was 5.33 ± 0.87. A slightly higher result was observed in the subscale “presence” (5.35 ± 1.14). The results of the research implemented during the period of severe psychological pressure associated with the COVID-19 pandemic provided information on symptoms of traumatic stress in the examined group of nurses. Their sense of security has been lowered and accompanied by an intensified reflection on issues concerning security. However, their current sense of meaning in life remains higher than the tendency to searching for it. The surveyed nurses received individual support mostly from significant others (i.e., other than family and friends). They see positive changes resulting from painful experiences related to the COVID-19 pandemic, which can be characterized by adaptation in the form of post-traumatic growth.

## 1. Introduction

Corona Virus Disease 2019 (COVID-19) is a highly infectious disease with a long incubation period caused by Severe Acute Respiratory Coronavirus 2 (SARS-COV-2) [1]. On 31 December 2019, the China Office of the World Health Organization (WHO) was notified of new pneumonia cases of unknown etiology occurring in Wuhan (Hubei Province) [2]. The outbreak was first identified at a seafood market in Wuhan, where wild animals, including bats, were sold illegally [3]. Since then, the virus has spread rapidly inside and outside China, becoming a global health emergency [4]. Due to the rapid spread of COVID-19 worldwide it was declared a pandemic on 11 March 2020 by the WHO. The first case of COVID-19 infection in Poland was recorded on 4 March 2020 (in western Poland) [5]. Since then, the number of infected people has increased steadily, resulting in the announcement of an epidemic by the Minister of Health on 20 March 2020. In response to the COVID-19 epidemic, state authorities have adopted several drastic civil protection measures, including closing the state’s borders. Those measures include: obligatory 14-day quarantine for people returning from abroad, working remotely, suspension of face-to-face teaching at schools and universities, limiting the number of people simultaneously present in shops, limiting the number of attendees in churches, the obligation to cover the nose and mouth and closure of some service providers (i.e., hairdressers, beauticians, etc.) and cultural venues (i.e., cinemas, theaters, exhibitions, etc.). As of 2 July 2020, there have been 35,146 confirmed cases and 1,492 deaths due to coronavirus in Poland. From the beginning of the epidemic in Poland until 3 June 2020, 2404 healthcare workers (HCW) became infected with COVID-19 [5,6]. According to the Ministry of Health, among those infected, there were 1659 nurses, 660 doctors, and 85 midwives. On 3 June, HCW-infected constituted 9.79% of all infected in Poland, i.e., almost one in ten suffering from COVID-19 worked in a hospital, clinic, or some other healthcare facility [7].

In the face of such a tremendous global challenge as the COVID-19 pandemic, HCWs are an indispensable resource for every country. Their health and safety are of key importance, not only to ensure the continuity and safety of care for infected people but also to control the number of infections [8]. Nurses constitute the most numerous professional group among medical personnel in Poland and worldwide. They play an essential role in preventing the spread of infections, controlling infections, and supporting patients in isolation [9]. As a result, they are exposed to the negative psychological consequences of a pandemic. Also, they are the first in the line of duty to come into contact with COVID-19 infected patients. Older adults and people with chronic comorbidities are at greater risk of complications and they have higher rates of mortality from COVID-19. Nursing teams are essential for managing this patient population. Nurses also play a vital role in providing public education concerning preventing and reducing the spread of the disease. Due to nurses’ unique nature, they require the necessary support [10].

The COVID-19 pandemic not only affects physical health but can also lead to mental health problems such as sleep problems, depression, and traumatic stress [11,12]. The United Nations have highlighted in their recommendations that HCWs are particularly vulnerable to the psychological effects of the COVID-19 pandemic [13]. Published evidence enumerates significant changes in the work environment and the requirements for HCWs, i.e., working under more significant pressure or an unfavorable work environment as causes of this vulnerability [14]. HCWs, besides the distress concerning the exposure to COVID-19, are concerned about the lack of personal protective equipment (PPE) or other necessary equipment, and the challenges of maintaining a family and providing care for children with an increase tendency to irregular working hours, increased workloads and anxiety when entering new clinical roles [15]. They often experience emotional tension and physical exhaustion from having to provide care to an increasing number of patients whose condition can deteriorate rapidly. Moreover, the HCW worker is also at risk of infection, death of co-workers or stigmatization and social exclusion due to work with infected patients [16,17]. There are also moral dilemmas related to making decisions regarding the provision of care with limited health care resources [18].

We lead an investigation concerning the level of posttraumatic stress, perceived social support, opinions on the positive and negative consequences of the epidemic, the sense of security, and the sense of meaning in life among nurses in a face of new and not fully understood epidemiological phenomena of a global nature. A literature search revealed that published evidence [2,8,10,11,12,14,16,18] has focused on determining the level of stress in HCWs in the context of the COVID-19 pandemic. However, there has been little research on perceived support, opinions on the positive and negative consequences of the epidemic, sense of security, and sense of meaning in life in the context of the pandemic COVID-19. Therefore, we were the first to take on the challenge of assessing those factors among nurses. The study aims to present the research protocol and preliminary results in assessing each of these focus areas among nurses in Poland during the COVID-19 pandemic.

## 2. Materials and Methods

### 2.1. Study Protocol

#### 2.1.1. Study Design

Our study has a cross-sectional design, and it was carried out between 1 May and 15 May 2020, among 325 nurses in Poland. After confirming the first case of COVID-19 in Poland and the recognition of the disease by WHO as a global pandemic on 20 March 2020, the Polish government introduced restrictions on face-to-face encounters and initiated home isolation measures. The resulting epidemiological consequences (the risk of infection of the interviewer and the respondent) and difficulties in a face to face contact with working nurses (government restrictions prohibiting direct contact of people outside the common household) influenced our decision to use computer-assisted web interviewing (CAWI). The method was practical and provided simplicity of questionnaire distribution in a situation of social isolation. The survey was conducted among nurses using ten “www” web pages addressed directly to them via the Facebook platform. The requests to complete the survey on the bulletin board were made twice during the surveyed time, i.e., on the first day of the survey and the 7th day of the survey. The provided link redirected prospective participants to the questionnaire was set up on the “Google Survey” portal. The completion of online questionnaires is an established method in healthcare research [19]. Participants were allowed to complete the survey only once. The study included the following stages as part of the research project: (1) Creating an electronic version of the questionnaire on google.com, (2) Providing a link to the questionnaire via Facebook fan pages addressed to nurses, (3) Statistical analysis of the research material received and preparation of publications and (4) Preparation of an educational module for nurses to shape the competences of dealing with traumatic work experiences in conditions of a high risk of coronavirus infection in pre- and postgraduate education of nurses.

#### 2.1.2. Ethics Approval

The Bioethics Committee issued ethical approval at the Medical University of Lublin (decision number: KE-0254/73/2020). The research was conducted following the ethical principles contained in Recommendations from the Association of Internet Researchers [20]. Participation in the study was voluntary and anonymous. All participants in the study gave their informed consent to participate in the study electronically. The informed consent form preceding the questionnaire’s questions contained an explanation of the purpose, subject of the research, the approximate duration of the study, and the method of filling out the questionnaire. After reading the information about the survey, the respondent was asked to express their willingness to participate in the survey by clicking “Yes” or withdrawing from the survey by closing the page in the web browser containing the survey or selecting the “No” option. Only those who chose “Yes” proceeded to the questionnaire page containing the questions. The respondent could resign from participation in the study at any time by closing the website with the questionnaire.

#### 2.1.3. Participants Eligibility

After giving informed consent to participate in the study, to verify the respondents, the next page reiterated information that the study was intended for nurses and included the following question: “Are you a nurse?” The respondent could answer “Yes” or “No.” If the answer was “No,” the questionnaire was closed automatically, expressing gratitude for the time spent. To be eligible for participation, respondents had to meet the following inclusion and exclusion criteria. The inclusion criteria included: (1) a nurse working during the COVID-19 epidemic defined as the period from 20 March 2020, (2) professional activity before the coronavirus epidemic during January and February 2020, and (3) providing informed consent to participate in the study through response “Yes”. The exclusion criteria included: (1) being on sick leave, maternity, parental or care to leave before the announcement of the epidemic in Poland (January and February 2020), (2) being on sick leave, maternity, parental or care leave during the announcement of the epidemic in Poland, (3) withdrawal from work for health reasons, and (4) refusal to give informed consent to participate in the study. There was no target recruitment size. As direct comparisons are not being drawn, a power calculation has not been performed.

### 2.2. Questionnaire

To achieve the objectives of the study, a structured questionnaire was developed, consisting of four standardized tools and a proprietary tool. In the instructions for each of the scales listed, the respondents were asked to provide ratings taking into account the current epidemiological situation. All scales used in the study were characterized by the optimal internal consistency (Table S1).

#### 2.2.1. Symptoms of Perceived Stress Related to Traumatic Stress

The authors used the Polish version of the Impact Event Scale-Revised (IES-R) by Weiss and Marmar to assess traumatic stress (PTSD), including those related to trauma, disturbing memories and persistent negative emotions resulting from the COVID-19 pandemic [21] in the Polish adaptation of Juczyński and Ogińska-Bulik [22]. The scale consists of 22 items describing the symptoms of perceived stress in the last seven days to the experienced traumatic event. Ratings are made on a 5-point Likert scale (from 0–4). It aims to establish the current, subjective feeling of discomfort related to specific events that have occurred. It captures three dimensions of PTSD: Intrusion (expressing recurring images, dreams, thoughts or perceptual sensations related to the trauma), Hyperarousal (characterized by increased alertness, medication, impatience, difficulty concentrating) and Avoidance (manifested by effort getting rid of thoughts, emotions, or conversations related to trauma). In the analysis of the obtained results, we adopted a more restrictive approach, justified by the current PTSD diagnosis criteria, that a diagnosis of PTSD can be suspected only in those who score above the cut-off point (>1.5) in the overall score in each of the three dimensions [22]. Moreover, the statements included in the scale were modified so that they indicate the current situation related to the COVID-19 epidemic. In 13 items of the scale, the COVID-19 epidemic was indicated as the element that should be assessed in a given statement.

#### 2.2.2. Perceived Social Support During the COVID-19 Pandemic

In order to assess the perceived adequacy of social support, the authors used the Multidimensional Scale of Perceived Social Support (MSPSS) by Zimet et al. [23] in the Polish adaptation of Buszman and Przybyły-Basista [24]. The scale consists of 12 items measured using a 7-point Likert scale, where 1 = “I strongly disagree”, and 7 = “I strongly agree”. The scale considers the multidimensionality of perceived social support, taking into account three basic sources of support (four statements for each source of support): significant other, family, and friends. The results can be calculated both for individual subscales and for the entire scale, obtaining an overall score for the degree of perceived adequacy of social support. It is assumed that the higher the result obtained by a respondent on the MSPSS scale, the higher the respondent perceives the level of social support is one’s environment [24].

#### 2.2.3. Assessment of the Positive and Negative Consequences of the COVID-19 Pandemic

In order to assess the consequences of the coronavirus epidemic, the authors used the shortened version of the Changes in Outlook Questionnaire (CIOQ) by Joseph et al. [25] in the Polish adaptation of Skalski [26]. The scale consists of 10 items, five pairs for positive and five for negative consequences. The respondent’s task is to answer them using the 6-point Likert scale (from 1 = “I strongly disagree” to 6 = “I strongly agree”), marking the extent to which the given statement applies to the respondent [26].

#### 2.2.4. Assessment of Safety Experience During the COVID-19 Pandemic

In order to assess the level of experienced safety, the authors employed the Safety Experience Questionnaire (SEQ) by Klamut [27]. The scale is an operationalization of the two-factor model, in which two subscales are distinguished: Sense of safety and Reflection on safety. The Sense of safety subscale examines the level of experienced safety related to the belief that basic needs are currently being met and that people have satisfactory living conditions and can function. Reflection on safety subscale examines the degree of taking into account matters related to personal safety, loved ones, the nation, and the world safety when assessing life situations and social reality. The scale consists of nine items (five for the Sense of Security subscale and four for the Reflection on safety subscale). The respondent’s task is to express an opinion on the 5-point Likert scale concerning the extent to which a given statement relates to one’s current situation (from 1 = “I strongly disagree” to 5 = “I strongly agree”) [27].

#### 2.2.5. Sense and Meaning of Life During the COVID-19 Pandemic

The Meaning in Life Questionnaire (MLQ) by Steger et al. assessed presence and search for meaning in life in our investigation [28]. We used its Polish adaptation by Kossakowska et al. [29] The MLQ questionnaire consists of 10 questions that the respondent can answer on a 7-point Likert scale (from “Absolute untruth” to “Absolute truth”). The questionnaire consists of two subscales: Presence and Search. The questionnaire assumes that each person uses their concept of sense/meaning/purpose in life. The Presence of meaning in life subscale measures present events, concerns, and the declared and obtained sense of meaning in life, in particular: understanding the meaning of the sense of life, having a clearly defined life goal, the sense of knowing what makes life meaningful, awareness of a satisfying goal in life and to deny the fact that an individual’s life has no purpose. The Searching for Meaning subscale, on the other hand, is concerned with evaluation from the perspective of the future and measures the need for a given person to search for and constantly give meaning and purpose in life. This subscale measures in particular: declaring the need to pursue sense, purpose, mission, meaning, and the need to give weight to one’s life [29].

#### 2.2.6. Sociodemographic Variables

In subsequent questions, the respondents were asked to provide set of sociodemographic data necessary for the statistical analysis of the collected material. The questions were concerned with the following variables: gender, age, marital status, place of residence, with whom the respondent lives, having children, education, completed postgraduate education, work experience, seniority in the nursing profession, a position held, whether in ones work the respondent cared for a suspected or diagnosed patient with SARS-CoV-2, whether the respondent participated in any training on the use of PPE and the functioning of medical facility during the COVID-19 pandemic, following by a request to self-assess knowledge about COVID-19 (ways of spreading, ways of protection, handling patient, etc.).

### 2.3. Statistical Analysis

The reliability of each questionnaire and its dimensions were assessed using Cronbach’s alpha coefficients of internal consistency (Table S1). Continuous variables are expressed as mean and standard deviation (SD) or median (interquartile range, IQR) as appropriate. The Shapiro-Wilk test was used to assess conformity with a normal distribution. The mean values of the scale used were compared between groups using a t-test (one-way analysis of variance ANOVA, in the case of at least three groups). Relationships between age, work experience, and scale were examined by Pearson correlation. The calculations and testing were performed using IBM SPSS Statistics for Windows, version 25 (IBM Corp., Armonk, NY, USA). *p*-values <0.05 were accepted as statistically significant.

## 3. Results

### 3.1. Characteristics of Participants

Table 1 presents the characteristics of the study group. The study involved 325 nurses working across Poland. The mean age was 39.18 ± 11.16 years. Most of the respondents lived in a city (66.46%; *n* = 216), were married (57.75%; *n* = 188) and lived with their family (67.69%; *n* = 220). The median length of employment as a nurse in the study group was 14 years (Q1 = 3; Q3 = 25). Among the surveyed nurses, 67.38% (*n* = 219), confirmed that they had received training on the COVID-19 epidemic. 46.46% (*n* = 151) of the surveyed nurses admitted that they nursed a patient diagnosed with COVID-19 or who was suspected of having the disease.

### 3.2. Symptoms of Traumatic Stress in the Studied Group of Nurses—IES-R Assessment Results

Table 2 shows the average overall score for the occurrence of traumatic stress and its three dimensions and their relationship with selected variables. The mean overall IES-R score in the study group was 1.78 ± 0.65. Among the dimensions of traumatic stress, the highest score was obtained in the Avoidance dimension 1.86 ± 0.73, then in the Hyperarousal 1.8 ± 0.78 and Intrusion 1.74 ± 0.83 dimensions. The overall result and the outcomes of each dimension are higher than 1.5 in the study group, therefore symptoms of traumatic stress can be recognized.

There were differences in the average values of experiencing traumatic stress in the Intrusion dimension in groups differentiated by marital status and having children. A higher score on this scale was observed in the respondents who were married and those with children (*p* < 0.05). A positive correlation was observed between work experience and the Intrusion dimension (*p* = 0.01). The other variables did not significantly differentiate the studied group in terms of experiencing traumatic stress related to the COVID-19 epidemic.

### 3.3. Perceived Social Support during the COVID-19 Pandemic—Results of the MSPSS Assessment

Table 3 presents the average values of the scales of perception of social support for three sources of support and the analysis of the relationship between the selected variables and the perceived level of support. In the studied group, the highest indicators of support for the study were obtained from other significant ones (22.58 ± 5.22), then from friends (21.91 ± 5) and family (21.45 ± 4.4).

A significant negative relationship between the support of friends and other people, and the age of the respondents was observed; higher nurses’ age correlated with the lower level of support they received from friends and others. Moreover, more support from the family was declared by people who were married and had children (*p* < 0.05). Childless respondents declared significantly greater support from friends (*p* = 0.004), while single people from family (*p* = 0.004).

### 3.4. Positive and Negative Consequences of the COVID-19 Pandemic—Results of the CIOQ Assessment

Table 4 presents the average scores concerning the positive and negative psychological changes associated with the COVID-19 epidemic and their relationship with selected variables. Among the surveyed nurses, higher mean values were noted in the subscale measuring positive psychological changes (18.56 ± 4.04).

A significant negative correlation was observed between age and the consequences of the epidemic in a negative dimension (r = −0.15; *p* = 0.007). Also, significantly more pronounced positive changes in the COVID-19 epidemic were observed in nurses living in rural areas and those who were married. On the other hand, a higher level of negative psychological changes related to the COVID-19 epidemic was found in respondents who are single, childless, undergraduate education, and those who completed a qualification course.

### 3.5. The Level of Experienced Safety During the COVID-19 Pandemic—Results of the SEQ Assessment

Table 5 presents the average results on scales measuring two aspects concerning the experienced safety and the analysis of selected demographic variables with Reflection on safety and its Sense of safety. In the study group, a higher average level of Reflection on the safety scale (4.21 ± 0.49) was observed.

Among the analyzed variables, postgraduate education significantly differentiated the study group on the Reflection on safety subscale. The respondents with a postgraduate diploma obtained a significantly higher level of evaluation in this subscale. Moreover, there was a significant positive relationship between the time of epidemic development and the level of the sense of safety, expressed in the Sense of safety subscale (*p* = 0.005).

### 3.6. The Sense of Meaning in Life During the COVID-19 Pandemic—Results of the MLQ Assessment

Table 6 presents the average results concerning the sense of meaning in life and searching for meaning in life, the general scale of the MLQ questionnaire, and the analysis of the relationship between them and selected sociodemographic variables. The mean MLQ score was 5.33 ± 0.87. A slightly higher result was observed in the Presence subscale (5.35 ± 1.14).

There was a significantly positive relationship between the Presence subscale and a negative relationship between the Search subscale and the surveyed nurses’ age. In the studied group, variables marital status and having children significantly differentiated the results on the Presence subscale. Married respondents and those with children exhibited a significantly higher level of sense of meaning in life.

## 4. Discussion

The outbreak of an epidemic poses an enormous challenge to the national health care system and the country’s economy, while the virus itself poses a threat to physical and mental health. For these reasons, the COVID-19 pandemic will have long-term consequences that will affect international and national public health policies [30]. In the circumstances of the COVID-19 pandemic, nurses are at the forefront of the fight against the virus worldwide. Nurses are actively involved in the fight against COVID-19, in a way that few professionals are, being required to demonstrate professionalism and to remain calm. However, many nurses experience fear of the unknown, stress, and concern for patients, themselves, colleagues, relatives, and friends [31]. The ubiquitous risk of COVID-19 infection may be the cause of psychosocial stress [32]. Therefore, the study aimed to present the preliminary results in terms of assessing the level of traumatic stress, received social support, the sense of positive and negative consequences of the epidemic, experiencing safety and sense of life in the group of nurses in Poland in the context of the COVID-19 pandemic. This study is part of more extensive research aimed at identifying the characteristics and psychological consequences of the COVID-19 pandemic among Poland’s professionally active nurses and factors potentially protecting against excessive stress. Summing up, the preliminary results indicate an intensification of traumatic stress symptoms with particularly pronounced symptoms of avoidance exhibiting in the studied group of nurses. Respondents characterized the experience of a pandemic by a reduced sense of security with an intense reflection on issues related to their personal safety, that of others, and the world. The nurses in the study received support mostly from significant others (i.e., other than family and friends). The respondents are characterized by a sense of the meaning in life; the tendency to seek it is less pronounced. They also notice positive changes in the existing situation, which may be an expression of adaptation in the form of posttraumatic growth.

Symptoms of posttraumatic stress appear after traumatic events beyond the scope of regular human experiences, such as a violent physical attack, torture, accidents, rape, or natural disasters [33]. Several studies have assessed the psychological effect during an epidemic, e.g., Severe Acute Respiratory Syndrome (SARS) and H_1_N_1_. Lu et al. [34] found that 17.3% of healthcare workers experienced significant psychological symptoms during the SARS epidemic when faced with the threat. On the other hand, Mak et al. [35] and Lam et al. [36] showed that more than 40% of people who survived the SARS epidemic experienced PTSD. Wu et al. [37] revealed that people who were isolated when working on wards treating patients with SARS or had relatives or loved ones who had contact with SARS exhibited a two to three times higher risk of developing PTSS symptoms, compared to those who were not exposed to the virus, whether professionally or among loved ones. The lower intensity of traumatic stress symptoms in the general population during the COVID-19 pandemic is confirmed by Forte et al. [38] by research conducted among Italian respondents. Participants exhibited a mean score of 22.38 on IES-R, with 1.01 on the Intrusion subscale, 1.06 on the Avoidance subscale, and 0.98 on the Hyperarousal subscale. Similar research lead amongst Egypt’s general population achieved the following mean results: overall score 34.25, Intrusion—13.68, Avoidance—12.83, and Hyperarousal—7.73 [39]. A similar average results indicating a mild stressful impact of the pandemic was obtained in the Chinese population in the research by Zhang and Ma [40] and Wang et al. [41]. Interesting research was conducted by Tan et al. [42] evaluating, among others, traumatic stress among medical workers (doctors and nurses) and non-medical workers (caregivers, pharmacists, technicians. administration, office workers and cleaners) in Singapore. The authors observed a lower mean IES-R score among healthcare workers during the COVID-19 epidemic than during the SARS epidemic, during which the severity of PTSD symptoms was three times higher than in the cited study. The authors suggested that respondents were more prepared for the COVID-19 epidemic, and they had in place infection control procedures following experiences from the previous SARS epidemic. The obtained results of our research indicated a high intensity of PTSD among the surveyed nurses, which may be due to the lack of experience in infection management because the last epidemic of an infectious disease in Poland before the COVID-19 occurred in 1963 and it was a smallpox epidemic covering the city of Wroclaw.

Most researchers concur concerning the salutary effect of social ties on an individual’s mental health and well-being [43]. Social support refers to the care and support people feel they receive from others [44]. In an epidemic situation, social support, apart from psychological support, reduces the adverse psychological effects of the epidemic [45]. Liu et al. [46] amongst a group of young adults in the US have proven a pivotal role of family support resulting in low levels of depression and lower PTSD symptoms during the COVID-19 pandemic. Xiao et al. [47] confirmed that in a group of doctors and nurses caring for COVID-19 patients in China, the level of anxiety, stress, and self-efficacy depended on social support.

Unfortunately, HCWs often neglect social relationships with friends or family due to heavy workloads or concerns about the risk of exposing others to disease as they come into contact with people with COVID-19. On the other hand, maintaining social contacts becomes a challenge due to the need to maintain social distance [18]. According to the results of studies from China, in the face of both the SARS epidemic in 2003 and the current COVID-19, residents of Hong Kong (2003) and Liaoning Province (2020) reported increased social and family support during the epidemic compared to the pre-epidemic period. Increased social support may result from the fact that many preventive measures have been introduced to avert the spread of the epidemic (i.e., closing workplaces, theaters, shopping centers, etc.) [40,48]. The cited body of literature included respondents from a general sample population, while in the presented research, they were explicitly nurses with the highest level of support obtained from the significant other. This may be since, despite the government’s restrictions in the form of closed workplaces, the introduction of online work, movement restrictions, etc., nurses were not subjected to those restrictions and continued their work. Due to the problematic situation they found themselves in; it can be assumed that they received the highest support from people in a similar position to them, i.e., colleagues from work who could relate to their concerns about the COVID-19 epidemic.

Experiencing a traumatic event has consequences for the psyche of an individual. One of the more severe effects of traumatic experiences is acute stress disorder (ASD) and post-traumatic stress disorder (PTSD). Despite the existence of negative consequences for an individual’s psyche, people experiencing a stress reaction may also declare some psychosocial benefits. This phenomenon is called posttraumatic growth (PTG), and it concerns life transformations as a result of attempts to deal with traumatic events. PTG is not regarded as an adaptation mechanism; however, as the result of adaptation. PTG is related to the use of remedial strategies. Development understood in this way may refer to changes in the philosophy of life, interpersonal contacts, or the deepening of self-understanding. It is estimated that 40–70% of people experience PTG due to experiencing a traumatic event [49]. The original research found a higher average in terms of positive psychological changes after the COVID-19 pandemic amongst participating nurses. The authors of the research, despite a thorough literature review, did not uncover any manuscripts examining the positive impact of the COVID-19 pandemic on psychological functioning. Therefore, we believe that these are the first results that describe this aspect.

Safety is one of the most critical categories allowing to describe the context of life and the way people function [50,51]. Safety is defined as an actual state of non-threat that is subjectively felt by individuals or groups [52]. In the light of the above definition, security should be understood in two perspectives: (1) objective, i.e., related to external, objective factors that are important for a proper life, and (2) subjective, i.e., individual, objective assessment of the state of possession or availability of essential goods relating to the individual experience. The feeling of security is significant; therefore, it constitutes a motivating factor either as a need [53] or as a value [54]. With the prospect of the COVID-19 pandemic, the sense of security for healthcare professionals may be low. The following factors may contribute to the low sense of security: close contact with patients infected or suspected of being infected, lack of personal protective equipment or insufficient number, lack of training on the subject of coronavirus, lack of procedures for caring for a patient with COVID-19, and finally little information about the virus itself. The sense of security may also be influenced by events that are not directly related to patient care but are consequences of the place where the individual works, e.g., fear of stigmatization, fear of infecting loved ones, or uncertainty about the direction of the epidemic and the consequences of forced social isolation. The results of the described original research demonstrated lower results in the Sense of security scale and higher in the Reflection on safety subscale. Therefore, in the studied group, reflection on the individual safety, loved ones, the nation, and the world has been intensified and accompanied by a reduced sense of security (understood as the belief that basic needs are met, that they have satisfactory working conditions and the ability to act). A sense of security during a pandemic is also enhanced by a well-prepared workplace, especially healthcare workers. According to the studies by Delegrado et al. [55] conducted among medical staff in Latin America, HCWs had limited access to personal protective equipment, and health authorities support during the COVID-19 pandemic, which may have affected their sense of security. Unfortunately, the authors of the study did not find studies evaluating the sense of security of medical personnel during the COVID-19 pandemic in the literature, so this is the first study to evaluate this issue.

Another critical issue in the pandemic context is the level of sense of meaning and meaning in life that health care workers experience in this challenging period. Different meanings (meaning) are assigned to events, places, persons, objects, and theories by every person [56]. Frankl believes that the meaning of life is a fundamental factor of human existence, and it primarily relates to the enormous existential power of a man to face adversities and everyday challenges [57,58]. Steger et al. [59] draw attention to the necessity to understand the time perspective in the studied dimensions of the presence of meaning in life and its search. According to the authors, the critical issue is to be aware of a goal in the present time and the need to pursue it and always acquire it. He defines this second dimension of meaning in life, i.e., seeking it as an activity related to constant effort, marked by persistence and intensity in establishing or expanding knowledge about the meaning of one’s own life. According to numerous studies, a high level of meaning in life increases resistance to stress and generally possesses a positive effect on physical health [60,61]. More studies confirmed the existence of a positive relationship between the meaning of life and the quality of life and well-being [62,63]. Also, associations between the meaning in life, high self-esteem and sense of control have been noticed [64]. The lack of meaning in life, on the other hand, leads to an internal emptiness and existential neurosis, presenting itself in the lack of willingness to live and act [57,65]. Likewise, there has been described a relationship between the lack of meaning in life and pathologies and disorders, abuse of addictive substances, and suicidal thoughts [66,67]. Trzebiński et al. [68] in their research concerning meaning in life and life satisfaction during COVID-19 outlined the following dependence of a mediated nature: basic hope supports meaning in life, and life satisfaction, and the increase in the latter two factors results in lower anxiety and COVID-19 stress. Original research described a slightly higher result in the Presence subscale as compared to the Search subscale. Those outcomes may result from the age of the respondents because, as shown by the research of Stager et al. [69], age is an important factor differentiating people in terms of the presence of meaning in life. Older people have a higher level of meaning in life than younger people, who, on the other hand, seek it more.

### 4.1. The Strengths and Limitations

The strengths of this research demand further consideration. Firstly, to the best of our knowledge, this is the first study assessing the psychological effects of the COVID-19 epidemic in a group of Polish nurses in such a multi-faceted manner. Secondly, our study implemented standardized questionnaires not yet used to assess psychosocial functioning in the context of the COVID-19 pandemic. Thirdly, our sample consists of nurses working in Poland who have never encountered an epidemic on the scale of COVID-19.

Nevertheless, our study also possesses some limitations. Firstly, our study is a cross-sectional study and does not show cause-effect or time-effect relationships. However, our results were descriptive rather than hypothesis-testing. Secondly, we adopted the strategy of distributing the questionnaire online due to the limitations of social contacts. Therefore it was not possible to collect data on people who refused to participate in the survey and no refusal rate was recorded. Moreover, when recruiting study participants, we relied on access to social networking sites, therefore, the surveyed population does not include participants who do not have access to social networking sites and nurses who do not use such sites. The above arguments make the generalization of our research results limited.

### 4.2. Implications and Future Directions

Our research’s preliminary results may provide a direction for the development of active protection programs for nurses against the adverse effects of the COVID-19 pandemic by strengthening protective factors for the pejorative phenomena related to it. A literature review revealed many gaps in research on certain psychological factors and their impact on healthcare professionals’ traumatic stress. Most of the published studies focus on traumatic stress or the impact of a pandemic on mental and physical health. Some elements such as social support, the sense of meaning and meaning in life, the positive and negative effects of a pandemic have not been thoroughly researched. Therefore, subsequent studies should be cross-sectional and broadly assessing the psychological context influencing the perception of traumatic stress among nurses and HCWs.

## 5. Conclusions

The preliminary results of conducted research in the period of intense psychological pressure related to the COVID-19 epidemic indicate that traumatic stress symptoms occur in the studied group of nurses. Their sense of security is lowered; they are accompanied by intense reflection on the issues concerning it. However, the sense of (currently felt) meaning in life remains higher than the tendency to seek it. The surveyed nurses perceive special support from the so-called significant other (other than family and friends). They look for positive changes resulting from painful experiences related to the COVID-19 pandemic, which may bear the hallmarks of adaptation in the form of posttraumatic growth.

## Figures and Tables

**Table 1:** Descriptive statistics of surveyed respondents (*n* = 325).

Characteristic	Group	Study Group
Age [years] ^b^		39.18 ± 11.16
Gender (female) ^a^		311 (96.7)
Place of residence (city) ^a^		216 (66,5)
Marital status (married) ^a^		188 (57.7)
Child(ren) in House (yes) ^a^		212 (65.2)
Living arrangements ^a^	Family	220 (67.7)
	Cohabitant	66 (20.3)
	Flat mate/roommate	11 (3.4)
	Alone	28 (8.6)
Education (Master’s degree or above) ^a^		142 (43.7)
Postgraduate education ^a^	Postgraduate diploma	43 (13.2)
	Specialist training course	145 (44.6)
	Qualification course	137 (42.2)
Years of experience [years] ^c^		14, (3–25)
Employment status (full-time) ^a^		317 (97.54)
Position ^a^	Nurse employed in the ward	247 (76)
	Ward nurse	44 (13.6)
	Head nurse	16 (4.9)
	Primary care nurse	18 (5.5)
Have you nursed a patient diagnosed with COVID-19 (yes) ^a^		151 (46.5)
Was there any training related to the coronavirus epidemic at work? (yes) ^a^		219 (67.4)
Knowledge concerning coronavirus ^a^	Very good	58 (17.8)
	Good	178 (54.8)
	Satisfactory	77 (23.7)
	Low	9 (2.8)
	Very low	3 (0.9)

Note: Data presented as: ^a^ *n* (%), ^b^ mean ± SD, or ^c^ median (Q1–Q3).

**Table 2:** The level of experienced traumatic stress and its components; selected sociodemographic variables.

Variable	Total Score		Intrusion		Avoidance		Hyperarousal	
	M ± SD	*p*	M ± SD	*p*	M ± SD	*p*	M ± SD	*p*
IES-R	1.78 ± 0.65		1.74 ± 0.83		1.86 ± 0.73		1.80 ± 0.78	
Age [years]	r = 0.2	0.717	r = 0.11	0.56	r = −0.07	0.21	r = −0.01	0.86
Place of Residence								
Urban area	1.78 ± 0.67	0.362	1.70 ± 8.83	0.161	1.87 ± 0.76	0.665	1.77 ± 0.82	0.271
Rural area	1.85 ± 0.60		1.84 ± 0.79		1.83 ± 0.69		1.87 ± 0.7	
Marital Status								
Married	1.85 ± 0.61	0.148	1.85 ± 0.61	0.047	1.85 ± 0.71	0.213	1.86 ± 0.71	0.147
Single	1.76 ± 0.7		1.61 ± 0.89		1.93 ± 0.78		1.77 ± 0.87	
Divorced/Separated/Widowed	1.63 ± 0.69		1.63 ± 0.81		1.67 ± 0.75		1.58 ± 0.85	
Child(ren) in House								
Yes	1.82 ± 0.61	0.539	1.81 ± 0.78	0.035	1.82 ± 0.72	0.189	1.81 ± 0.72	0.778
No	1.77 ± 0.71		1.61 ± 0.89		1.93 ± 0.76		1.79 ± 0.89	
Education								
Bachelor’s degree	1.82 ± 0.63	0.522	1.79 ± 0.81	0.234	1.87 ± 0.74	0.756	1.80 ± 0.76	0.955
Master’s degree or above	1.77 ± 0.67		1.68 ± 0.84		1.84 ± 0.73		1.80 ± 0.81	
Years of experien [years]	r = 0.062	0.271	r = 0.144	0.01	r = −0051	0.363	r = 0.036	0.052
Completed Postgraduate Education								
Postgraduate diploma	1.72 ± 0.65	0.433	1.72 ± 0.65	0.888	1.69 ± 0.74	0.76	1.78 ± 0.79	0.666
Specialist training course	1.78 ± 0.66		1.75 ± 0.84		1.81 ± 0.74		1.77 ± 0.77	
Qualification course	1.85 ± 0.63		1.76 ± 0.81		1.95 ± 0.72		1.85 ± 0.79	
Was There Any Training Related to the Coronavirus Epidemic at Work?								
Yes	1.80 ± 0.65	0.982	1.73 ± 0.80	0.725	1.87 ± 0.74	0.784	1.81 ± 0.76	0.916
No	1.80 ± 0.64		1.77 ± 0.86		1.84 ± 0.73		1.80 ± 0.83	
Variable	Total score		Intrusion		Avoidance		Hyperarousal	
	M ± SD	*p*	M ± SD	*p*	M ± SD	*p*	M ± SD	*p*
Have You Nursed a Patient Diagnosed with COVID-19								
Yes	1.84 ± 0.71	0.234	1.82 ± 0.89	0.108	1.86 ± 0.76	0.989	1.86 ± 0.84	0.246
No	1.76 ± 0.58		1.68 ± 0.75		1.86 ± 0.71		1.76 ± 0.72	
The development of the epidemic [days]	r = −0.412	0.459	r = −0.067	0.226	r = 0.029	0.6	r = −0.054	0.332

Key: M: mean; SD: standard deviation; r: correlation coefficient; IES-R: Impact Event Scale-Revised.

**Table 3:** The level of perceived social support during the pandemic and selected sociodemographic variables.

Variable	Family		Friends		Others	
	M ± SD	*p*	M ± SD	*p*	M ± SD	*p*
MSPSS	21.45 ± 4.40		21.91 ± 5.0		22.58 ± 5.22	
Age [years]	r = 0.07	0.19	r = −0.17	0.002	r = −0.14	0.01
Place of Residence						
Urban area	21.53 ± 5.54	0.702	21.99 ± 4.98	0.657	22.85 ± 5.27	0.184
Rural area	21.28 ± 5.14		21.73 ± 5.06		22.04 ± 5.08	
Marital Status						
Married	22.27 ± 4.61	0.004	21.57 ± 5.10	0.058	22.53 ± 5.09	0.542
Single	20.14 ± 6.46		22.83 ± 4.82		22.92 ± 5.59	
Divorced/Separated/Widowed	20.85 ± 5.23		20.91 ± 4.69		21.79 ± 4.71	
Child(ren) in House						
Yes	21.88 ± 5.04	0.048	21.32 ± 5.04	0.004	22.33 ± 4.92	0.240
No	20.64 ± 6.43		23.01 ± 4.76		23.04 ± 5.71	
Education						
Bachelor’s degree	21.65 ± 5.01	0.44	21.92 ± 5.06	0.966	22.53 ± 5.04	0.866
Master’s degree or above	21.18 ± 5.88		21.89 ± 4.94		22.63 ± 5.44	
Work experience [years]	r = 0.061	0.279	r = −0.099	0.078	r = −0.104	0.065
Completed Postgraduate Education						
Postgraduate diploma	21.70 ± 5.61	0.835	21.58 ± 5.34	0.409	22.65 ± 5.54	0.061
Specialist training course	21.56 ± 4.94		21.59 ± 4.83		21.85 ± 5.33	
Qualification course	21.24 ± 5.82		22.34 ± 5.07		23.32 ± 4.91	
Was There Any Training Related to the Coronavirus Epidemic at Work?						
Yes	21.47 ± 5.35	0.891	21.84 ± 5.04	0.727	22.55 ± 5.17	0.898
No	21.38 ± 5.52		22.05 ± 4.93		22.63 ± 5.33	
Have You Nursed a Patient Diagnosed with COVID-19?						
Yes	21.71 ± 5.26	0.415	22.02 ± 2.26	0.707	22.63 ± 5.82	0.871
No	21.22 ± 5.32		21.81 ± 4.77		22.53 ± 4.64	
The development of the epidemic [days]	r = 0.07	0.202	r = 0.06	0.278	r = −0.005	0.929

Key: The abbreviations correspond to the Table 2; MSPSS: Multidimensional Scale of Perceived Social Support.

**Table 4:** The level of positive and negative consequences of the pandemic and selected sociodemographic variables.

Variable	Positive Change		Negative Change	
	M ± SD	*p*	M ± SD	*p*
CIOQ	18.56 ± 4.04		14.28 ± 4.49	
Age [years]	r = 0.04	0.183	r = −0.15	0.007
Place of Residence				
Urban area	18.55 ± 4.06	0.046	14.12 ± 14.59	0.368
Rural area	19.49 ± 3.94		14.60 ± 4.28	
Marital Status				
Married	19.41 ± 3.87	0.016	13.67 ± 3.96	0.006
Single	18.19 ± 4.45		15.42 ± 5.27	
Divorced/Separated/Widowed	17.91 ± 3.08		14.12 ± 4.14	
Child(ren) in House				
Yes	19.09 ± 3.74	0.166	13.87 ± 3.88	0.249
No	17.44 ± 4.53		15.04 ± 5.40	
Education				
Bachelor’s degree	18.87 ± 3.86	0.995	14.85 ± 4.57	0.009
Master’s degree or above	18.87 ± 4.27		13.54 ± 4.3	
Work experience [years]	r = 0.027	0.636	r = −0.086	0.125
Completed Postgraduate Education				
Postgraduate diploma	19.25 ± 4.30	0.422	14.0 ± 4.47	<0.001
Specialist training course	18.54 ± 4.16		13.28 ± 4.27	
Qualification course	19.09 ± 3.82		15.42 ± 4.50	
Was There Any Training Related to the Coronavirus Epidemic at Work?				
Yes	19.09 ± 3.76	0.160	14.08 ± 4.47	0.244
No	18.41 ± 4.53		14.70 ± 4.54	
Have You Nursed a Patient Diagnosed with COVID-19?				
Yes	19.07 ± 4.41	0.410	14.50 ± 5.07	0.405
No	18.69 ± 3.69		14.08 ± 3.93	
The development of the epidemic [days]	r = 0.017	0.756	r = −0.035	0.526

Key: The abbreviations correspond to the Table 2; CIOQ: Changes in Outlook Questionnaire.

**Table 5:** The level of experiencing security and selected sociodemographic variables.

Variable	Sense of Safety		Reflection on Safety	
	M ± SD	*p*	M ± SD	*p*
SEQ	3.23 ± 0.79		4.21 ± 0.49	
Age [years]	r = −0.02	0.723	r = 0.03	0.612
Place of Residence				
Urban area	3.21 ± 0.81	0.496	4.21 ± 0.51	0.699
Rural area	3.27 ± 0.74		4.23 ± 0.44	
Marital Status				
Married	3.24 ± 0.76	0.76	4.26 ± 0.42	0.413
Single	3.27 ± 0.84		4.15 ± 0.60	
Divorced/Separated/Widowed	3.06 ± 0.76		4.17 ± 0.41	
Child(ren) in House				
Yes	3.19 ± 0.75	0.201	4.24 ± 0.42	0.209
No	3.31 ± 0.86		4.17 ± 0.59	
Education				
Bachelor’s degree	3.22 ± 0.79	0.842	4.21 ± 0.45	0.913
Master’s degree or above	2.24 ± 0.79		4.21 ± 0.53	
Work experience [years]	r = −0.056	0.322	r = 0.014	0.808
Completed Postgraduate Education				
Postgraduate diploma	3.24 ± 0.84	0.871	4.39 ± 0.40	0.02
Specialist training course	3.20 ± 0.83		4.15 ± 0.51	
Qualification course	3.25 ± 0.72		4.22 ± 0.49	
Was There Any Training Related to the Coronavirus Epidemic at Work?				
Yes	3.27 ± 0.74	0.153	4.21 ± 0.50	0.808
No	3.14 ± 0.87		4.22 ± 0.46	
Have You Nursed a Patient Diagnosed with COVID-19?				
Yes	3.22 ± 0.83	0.971	4.19 ± 0.56	0.471
No	3.23 ± 0.75		4.23 ± 0.41	
The development of the epidemic [days]	r = 0.157	0.005	r = −0.092	0.098

Key: The abbreviations correspond to the Table 2; SEQ: Safety Experience Questionnaire.

**Table 6:** The level of experiencing security and selected sociodemographic variables.

Variable	Total Score		Presence Subscale		Search Subscale	
	M ± SD	*p*	M ± SD	*p*	M ± SD	*p*
MLQ	5.33 ± 0.87		5.35 ± 1.14		5.30 ± 0.94	
Age [years]	r = 0.03	0.612	r = 0.14	0.011	r = −0.11	0.44
Place of Residence						
Urban area	5.31 ± 0.9	0.543	5.32 ± 1.2	0.44	5.3 ± 0.98	0.848
Rural area	5.37 ± 0.8		5.43 ± 1.01		5.32 ± 0.85	
Marital Status						
Married	5.43 ± 0.83	0.808	5.55 ± 1.02	<0.0001	5.3 ± 0.9	0.122
Single	5.18 ± 0.94		4.96 ± 1.29		5.4 ± 0.96	
Divorced/Separated/Widowed	5.23 ± 0.81		5.45 ± 1.02		5.01 ± 1.04	
Child(ren) in House						
Yes	5.39 ± 0.81	0.80	5.53 ± 1.02	0.0001	5.25 ± 0.88	0.175
No	5.21 ± 0.96		5.03 ± 1.28		5.4 ± 1.04	
Education						
Bachelor’s degree	5.28 ± 0.84	0.262	5.29 ± 1.05	0.265	5.27 ± 0.92	0.466
Master’s degree or above	5.39 ± 0.9		5.43 ± 1.24		5.35 ± 0.96	
Work experience [years]	r = 0.007	0.901	r = 0.092	0.1	r = −0.099	0.08
Completed Postgraduate Education						
Postgraduate diploma	5.44 ± 0.76	0.654	5.54 ± 0.99	0.106	5.34 ± 0.83	0.173
Specialist training course	5.32 ± 0.87		5.44 ± 1.13		5.2 ± 0.95	
Qualification course	5.3 ± 0.91		5.2 ± 1.18		5.4 ± 0.95	
Was There Any Training Related to the Coronavirus Epidemic at Work?						
Yes	5.33 ± 0.85	0.871	5.36 ± 1.13	0.898	5.31 ± 0.88	0.884
No	5.32 ± 0.91		5.34 ± 1.17		5.29 ± 1.05	
Have You Nursed a Patient Diagnosed with COVID-19?						
Yes	5.35 ± 0.83	0.732	5.31 ± 1.11	0.558	5.38 ± 0.86	0.177
No	5.31 ± 0.9		5.39 ± 1.17		5.24 ± 0.99	
The development of the epidemic [days]	r = −0.069	0.214	r = −0.048	0.392	r = −0.07	0.205

Key: The abbreviations correspond to the Table 2; MLQ: Meaning in Life Questionnaire.

## Supplementary Materials

The following are available online at https://www.mdpi.com/1660-4601/17/18/6491/s1, Table S1: Internal consistency of used questionnaires.
