# Supplementary material for: The Severity of Traumatic Stress Associated with COVID-19 Pandemic, Perception of Support, Sense of Security, and Sense of Meaning in Life among Nurses: Research Protocol and Preliminary Results from Poland
Source: Int J Environ Res Public Health. 2020 Sep 7;17(18):6491. doi: 10.3390/ijerph17186491 (PMC7559728; doi:10.3390/ijerph17186491)
Supplement: Supplementary file 1 [file ijerph-17-06491-s001.pdf]

International Journal of
Environmental Research and Public Health

MDPI

Supplementary Materials

The Severity of Traumatic Stress Associated with COVID-19 Pandemic, Perception of Support, Sense of Security, and Sense of Meaning of Life Among Nurses: Research Protocol and Preliminary Results from Poland

Grzegorz Józef Nowicki [1,*], Barbara Ślusarska [1], Kinga Tucholska [2], Katarzyna Naylor [3], Agnieszka Chrzan-Rodak [1] and Barbara Niedorys [1]

Table 1S. Internal consistency of used questionnaires.

	alfa **Cronbach**
IES-R Total score	0.86
IES-R Intrusion	0.86
IES-R Avoidance	0.74
IES-R Hyperarousal	0.80
MSPSS Family	0.90
MSPSS Friends	0.92
MSPSS Other	0.90
CIOQ Positive change	0.72
CIOQ Negative change	0.78
SEQ Sense of safety	0.84
SEQ Reflection on safety	0.71
MLQ Total score	0.82
MLQ Presence subscale	0.85
MLQ Search subscale	0.71

Key: M: IES-R: Impact Event Scale-Revised; MSPSS: Multidimensional Scale of Perceived Social Support; CIOQ: Changes in Outlook Questionnaire; SEQ: Safety Experience Questionnaire; MLQ: Meaning in Life Questionnaire.
